# Translation and validation of two disease-specific patient-reported outcome measures (Bladder Cancer Index and FACT-Bl-Cys) in Dutch bladder cancer patients

**DOI:** 10.1186/s41687-019-0149-7

**Published:** 2019-09-14

**Authors:** Charlotte T. J. Michels, Carl J. Wijburg, Inger L. Abma, J. Alfred Witjes, Janneke P. C. Grutters, Maroeska M. Rovers

**Affiliations:** 1 Department of Urology, Rijnstate Hospital, Arnhem, Netherlands; 2 Department of Operating Rooms, Radboud Institute for Health Sciences, Radboud University Medical Center, Nijmegen, Netherlands; 3 IQ healthcare, Radboud Institute of Health Sciences, Radboud University Medical Center, Nijmegen, Netherlands; 4 Department of Urology, Radboud University Medical Center, Nijmegen, Netherlands; 5 Department for Health Evidence, Radboud Institute of Health Sciences, Radboud University Medical Center, Nijmegen, Netherlands

**Keywords:** Bladder cancer, Radical cystectomy, Patient-reported outcomes measures, Psychometrics, Validity, Reliability, Responsiveness

## Abstract

**Background:**

The Bladder Cancer Index (BCI) and Functional Assessment of Cancer Therapy-Bladder-Cystectomy (FACT-Bl-Cys) were developed to measure disease-specific health-related quality of life (HRQOL) in bladder cancer patients and patients treated with radical cystectomy, respectively. Both patient-reported outcome measures (PROMs) are frequently used in clinical practice, but are not yet validated according to the COSMIN criteria and not yet available in Dutch. Therefore, the aim of this study was to translate the BCI and FACT-Bl-Cys into Dutch and to evaluate their measurement properties according to the COSMIN criteria.

**Methods:**

The BCI and FACT-Bl-Cys were translated into Dutch using a forward-backward method, and subsequently administered at baseline (pre-operatively) and 3 months post-operatively in bladder cancer patients who received a radical cystectomy. Validity (content and construct), reliability (internal consistency, test-retest reliability, and measurement error), floor and ceiling effects, and responsiveness were assessed according to the COSMIN criteria.

**Results:**

Forward-backward translation encountered no particular linguistic problems. In total 260 patients completed the baseline measurement, while 182 patients completed the three-month measurement. Only a ceiling effect was identified for the BCI. Hypotheses testing for construct validity was satisfying, as 67% and 92% of the hypothesized correlations were confirmed. Structural validity was moderate for both measures, as confirmatory factor analyses showed limited fit. Reliability of both PROMs was good. The intraclass correlation coefficient (ICC) of the BCI domains ranged from 0.47 to 0.93, minimal value of Cronbach’s α was 0.70, smallest detectable change on group level (SDC group) ranged from 1.9 to 8.6. The ICC of the FACT-Bl-Cys domains ranged from 0.43 to 0.83, minimal value of Cronbach’s α was 0.77, SDC group was around 1. Only the FACT-Bl-Cys total score was found to be responsive to changes in generic quality of life.

**Conclusions:**

The Dutch versions of the BCI and FACT-Bl-Cys were shown to be reliable and have good content validity. Structural validity was limited for both measures. Only the FACT-Bl-Cys total score was responsive to changes in generic HRQOL. Despite some limitations, both PROMs seem suitable for use in clinical practice and research.

**Electronic supplementary material:**

The online version of this article (10.1186/s41687-019-0149-7) contains supplementary material, which is available to authorized users.

## Introduction

Bladder cancer (BCa) ranks ninth in worldwide cancer incidence and is one of the most expensive malignancies to manage [1, 2]. The spectrum of BCa includes non-muscle-invasive, muscle-invasive, and metastatic disease, each with its own clinical behavior, prognosis, and treatment.

Due to the heterogeneous BCa population, the various treatments (e.g. transurethral surgery, chemotherapy, radical cystectomy), and the lack of long-term follow-up data, not much is known regarding the patient burden imposed by BCa [3–5]. Since BCa patients will usually undergo several treatments, measuring health-related quality of life (HRQOL) with valid instruments is important for clinicians and patients when making informed decisions about treatments, based on patients’ experiences [6, 7]. Increasingly more clinical trials [8] and comparative effectiveness studies [9] include patient-reported outcomes (PROs), in addition to objective outcomes.

In 2016, a systematic review was published in which Danna et al. [10] advised to use the Functional Assessment of Cancer Therapy-Bladder-Cystectomy (FACT-Bl-Cys, formerly known as the FACT-VCI) [11, 12] when studying HRQOL in patients undergoing radical cystectomy and to use the Bladder Cancer Index (BCI) [13, 14] when studying HRQOL in patients with nonmuscle-invasive or muscle-invasive BCa disease of any stage. Authors report that the FACT-Bl-Cys was the only available measure for muscle-invasive BCa patients and the BCI has unique “bother” scales, which quantifies the symptoms’ impact [10]. Both patient-reported outcomes measures (PROMs) are frequently used and have been translated into various languages (i.e. BCI is available in Spanish, French, Hungarian [15–17], and FACT-Bl-Cys is available in Korean and Swedish [18, 19]), but were not yet available in Dutch. In 2018, Mason et al. revealed in a systematic review that most PROMs related to BCa had limited information reported on their measurement properties [4]. The FACT-Bl-Cys and BCI are suggested to be most promising, but some measurement properties (e.g. content validity, measurement error, and responsiveness) are still unknown [4].

To assess whether both PROMs provide valuable information in clinical practice and research, it is warranted to study their measurement properties. The COnsensus-based Standards for the selection of health Measurement INstruments (COSMIN) checklist was developed to assess the quality of validation studies and can be used to design and report on these studies [20–22]. The aim of this study was to translate the BCI and FACT-Bl-Cys into Dutch and to evaluate the validity, reliability, floor and ceiling effects, and responsiveness according to the COSMIN criteria.

## Methods

### Translation procedure

**Fig. 1:**
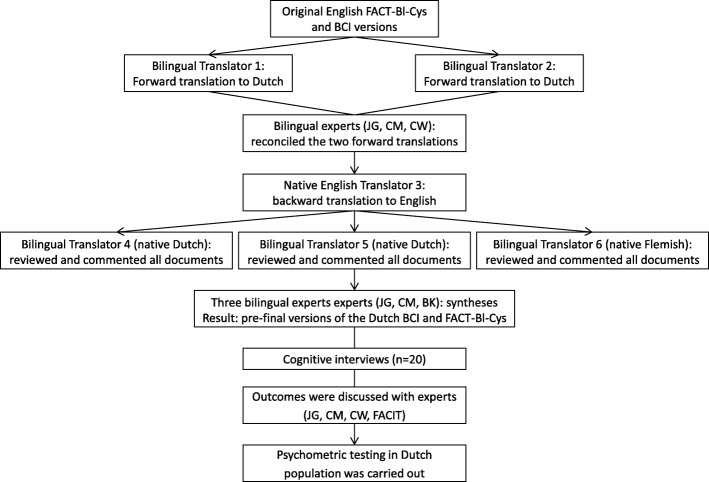
Translation procedure

The BCI and FACT-Bl-Cys were translated into Dutch, using a forward–backward method according to published guidelines, involving six steps [23, 24]. A schematic overview of the translation procedure is presented in Fig. 1. First, two bilingual native Dutch translators independently translated the United States English versions of the BCI and FACT-Bl-Cys into Dutch (forward translation). Second, two independent native Dutch speakers (JG, CM) reconciled the two forward translations into one forward translation. Any uncertainties were discussed among JG, CM and a urologist (CW), and resolved by consensus. Third, one independent native English translator, who is fluent in Dutch, translated the forward translation back to English. The translator was uninformed of the concepts explored and blinded to the original instruments. Fourth, three independent bilingual experts (two native Dutch and one native Flemish speaker, all fluent in English) reviewed and commented on all documents (original version, forward and backward translations). The synthesis process was carefully documented and outcomes were evaluated by three Dutch speakers (a language coordinator, JG, CM). Conceptual rather than literal translation was leading, aiming to preserve the original meaning of each item. This resulted in pre-final versions of the Dutch BCI and FACT-Bl-Cys. Fifth, both pre-final versions were tested during a pilot study, in which cognitive interviews were carried out among 20 BCa patients who underwent a radical cystectomy. Patients completed both pre-final versions and were interviewed about the comprehension of items and the chosen response, according to Functional Assessment of Chronic Illness Therapy (FACIT) guideline and instructions [24]. Patients were randomly recruited from Radboud university medical center (Nijmegen, the Netherlands) and Rijnstate Hospital (Arnhem, the Netherlands). Finally, experts (JG, CM, CW, and FACIT representatives) discussed the outcomes and final versions were realized to enable the evaluation of their psychometrics.

### Study design and sample

As part of the RACE study (trial identifying number NTR5362, Dutch Trial Registry, www.trialregister.nl) patients were asked to complete four measures (i.e. BCI, FACT-Bl-Cys, EQ-5D-5 L, and EQ-VAS). Patients that completed the three-month follow-up period were included in this substudy. RACE is a comparative effectiveness study aiming to determine the (cost-)effectiveness of robot-assisted and open radical cystectomy [25]. Radical cystectomy is the standard treatment of non-metastatic, invasive BCa and is curative in the majority of patients with localized disease [1]. This surgery is associated with a high complication rate, varying between 49% and 68% [26]. Studies suggest that radical cystectomy affects urinary, sexual and bowel function, and body image which can lead to anxiety and depression [27–30].

All participants in this validation study fulfilled the inclusion criteria of the RACE study: they were 18 years or older, had an oncological indication for radical cystectomy, a histologically proven primary muscle invasive urothelial carcinoma or therapy resistant high-risk non muscle-invasive BCa (CIS, refractair pTa-1), a non-metastatic tumor (cT1a-cT4a, cN0M0), they were able to complete Dutch questionnaires, and provided written informed consent. Patients were excluded from RACE when they met one of the following criteria: previous major abdominal surgery (i.e. existing stomata, low anterior resection of the rectum or rectal amputation, status after open aortabifemoral graft, status after right hemicolectomy), pregnancy, morbid obesity (BMI ≥ 40 kg/m^2^), radical cystectomy performed in combination with a nephrectomy or a partial colon resection.

Measures used in this validation study were the translated Dutch versions of the BCI and FACT-Bl-Cys, and the validated Dutch versions of the EuroQol 5-Domain 5-Level (EQ-5D-5 L) [31] and the EuroQol Visual Analogue Scale (EQ-VAS). Participants could choose to complete the PROMs on paper or electronically. Participants completed the PROMs at baseline (T0, pre-operatively) and 3 months post-operatively (T3). Patients who completed the three-month measurement (T3) were asked to complete the PROMs again within 2 weeks (T3 retest).

### Measures

#### Bladder Cancer index (BCI)

The BCI is developed to measure disease-specific HRQOL in patients with nonmuscle-invasive and muscle-invasive BCa disease of any stage. It consists of 36 items covering three domains: urinary (14 items), bowel (10 items), and sexual (12 items) [13, 14]. Each domain consists of two subdomains (function and bother). Item responses are based on 4- or 5-point Likert scales; domain and subdomain scores are standardized to a 0–100 point scale where higher scores indicate better HRQOL. No total BCI score is calculated. Function items focus on the frequency of the disease symptoms and bother items reflect the individual perception of these symptoms. According to BCI scoring instructions, the number of non-missing items needed to compute domain scores varied from 10 (urinary domain and sexual domain) to 4 (“urinary function”, “bowel function”, “sexual bother”), otherwise scores were set to missing.

#### Functional assessment of Cancer therapy-bladder-cystectomy (FACT-Bl-Cys)

The FACT-Bl-Cys is developed to measure condition-specific HRQOL in patients treated with radical cystectomy. It consists of the four general FACT-General (FACT-G) [32] domains (physical, social/family, emotional, and functional well-being) plus one additional Bl-Cys domain (17 items) covering urinary, sexual and bowel function, and body image [11, 12]. Thus, the FACT-Bl-Cys measure consists of five domains (physical, social/family, emotional, functional well-being, and Bl-Cys). The measure consists of 44 items and responses are based on 5-point Likert scales, with higher scores indicating better HRQOL. The total FACT-Bl-Cys score can range from 0 to 168. According to FACT-Bl-Cys scoring instructions, more than 50% of the items per domain need to be answered in order to calculate a domain score.

### Statistical analyses

COSMIN recommendations were used as a guide for evaluating the measurement properties of the Dutch BCI and FACT-Bl-Cys [22]. Validity, reliability, floor and ceiling effects, and responsiveness were assessed as described below. Analyses were performed using IBM SPSS Statistics software (version 22.0, IBM Corp., Armonk, NY, USA). Additionally, R version 3.5.2 (R Foundation for Statistical Computing, Vienna, Austria), including the packages mice (version 3.5.0) [33] and SemTools (version 0.5–1.928) [34] was used for multiple imputation and confirmatory factor analyses (CFA).

Because the BCI is developed for generic BCa patients, we determined its measurement properties at baseline (T0, pre-operatively), with the exception of test-retest and measurement error which were based on T3 and T3 retest. The test-retest and measurement error could not be assessed at T0, because there was no possibility to distribute and complete the PROMs again within two weeks since patients received a cystectomy on short term after T0. Because the FACT-Bl-Cys is developed for patients treated with radical cystectomy, we determined its measurement properties after the cystectomy had been performed, i.e. at T3 and T3 retest.

#### Floor and ceiling effects

Floor and ceiling effects are considered to be present if more than 15% of respondents achieved the lowest or highest possible score, respectively [21, 35]. We evaluated the presence of floor and ceiling effects of single scores in this study to assess interpretability. Interpretability [20] is the degree to which one can assign a qualitative meaning on a quantitative score or change in score. Additionally, floor and ceiling effects can also impact the responsiveness (if scores cannot further improve or deteriorate) and validity of an instrument (as patients with the lowest or highest possible score cannot be distinguished from each other)*.*

#### Validity

Validity in this study was assessed by content validity and construct validity [20]. Content validity was evaluated from the perspective of researchers, patients, and urologists. First, the component of content validity known as face validity was determined by discussing with the research team whether the two measures gave the impression of adequately reflecting the construct to be measured. Second, content validity was evaluated during the above described cognitive interviews (*n* = 20). An interview guide was used for the interviews and respondents were invited to appraise the two measurement instruments on relevance, comprehensiveness, and comprehensibility [36]. Input from the interviews was summarized and notable similarities and differences in opinion among respondents were registered and closely evaluated in our research team.

Construct validity was evaluated by structural validity and hypotheses testing. First, structural validity was assessed by confirmatory factor analyses (CFA) to confirm the previously suggested dimensionality of both PROMs [37]. Regarding BCI, the original validation study [13] identified three primary domains (urinary, bowel and sexual) and one translation study [16] reported that data fitted to six subdomains (function and bother). Therefore, in this study BCI items were hypothesized to load on three or six factors. Regarding FACT-Bl-Cys, the original validation study [11] identified three factors in the Bl-Cys domain and one translation study [18] reported that the Bl-Cys domain fitted on three other factors. Therefore, the FACT-G (four domains: physical, social/family, emotional, functional well-being) was hypothesized to load on four factors and the individual Bl-Cys domain on two different three-factor structures. Which items are structured within which factor is presented in Table 4.

For a CFA, COSMIN advises to use a sample size of 7 patients per item, with a minimum number of 100 patients [38]. Since our sample size was too small according to the COSMIN criteria as cases with missing items are not analyzed in a CFA (i.e. listwise deletion), we performed multiple imputation by chained equations generating 25 independent imputation datasets. Data were imputed at item level using a predictive mean matching approach. CFA was performed on the imputed datasets. Model fit was evaluated based on the Comparative Fit Index (CFI), Tucker-Lewis index (TLI), Root Means Square Error of Approximation (RMSEA), and Standardized Root Mean Square Residual (SRMR). The criteria for unidimensionality include CFI ≥ 0.95, TLI ≥ 0.95, RMSEA≤0.06, and SRMR≤0.08 [39].

Second, we tested various hypotheses by analyzing the association between the BCI domains and FACT-Bl-Cys domains, using Pearson’s correlation coefficients. Coefficients were considered low (< 0.30), moderate (0.30–0.69) or high (≥0.70). If ≥75% of the set number of hypotheses were confirmed, the construct validity was considered good [21]. Pre-specified hypotheses regarding BCI at T0 were that: a) correlations among different 3 domains (urinary, bowel, sexual) are low as they measure different constructs; b) correlations between the BCI domains and FACT-G, EQ-5D-5 L, EQ-VAS are moderate, due to differences between generic and disease-specific instruments. Pre-specified hypotheses regarding FACT-Bl-Cys at T3 were that: a) correlations among 5 different domains (physical, social/family, emotional, functional, Bl-Cys) are moderate based on original validation studies [11, 40]; b) correlation between the Bl-Cys domain and EQ-5D-5 L is moderate; c) correlation between FACT-Bl-Cys total score and the EQ-5D-5 L and EQ-VAS is moderate, due to differences between generic and disease-specific instruments. For the BCI and FACT-Bl-Cys there were 12 and 13 pre-specified hypotheses formulated, respectively.

#### Reliability

Reliability was assessed by analyzing internal consistency, test-retest reliability, and measurement error [20].

Internal consistency [20] of subscale items was measured using Cronbach’s α, to assess the degree of interrelatedness among the items of (sub) scales of the instrument. Values between 0.70 and 0.95 are considered adequate [21, 41].

Test-retest reliability [20] was assessed by calculating the intraclass correlation coefficient (ICC), using a two-way random effects model to compute absolute agreement (single measures) [21, 42]. Values ≥0.70 are considered to be reliable [21]. Patients who completed the three-month measure were assumed to be in a stable physical state and were asked to complete the PROMs again after 2 weeks.

Measurement error [20] was assessed by calculating the standard error of measurement (SEM) using the formula: √(σ^2^_o_ + σ^2^_residual_) [22, 43]. Additionally, the smallest detectable change on group level (SDC group) was calculated from the SDC at individual level (SDC_individual_ = 1.96*√2*SEM), using the formula: SDC_individual_/√n [44].

#### Responsiveness

Responsiveness should be determined in a population that shows actual change [20]. First, we assessed the change scores of both measures between T0 and T3. Second, we evaluated the occurrence of complications (defined as grade 1 to 5 according to the Clavien-Dindo classification) in relation to the change scores of both measures between T0 and T3. Third, we assessed the correlation and effect size of change score of both measures with the change score of the EQ-VAS between T0 and T3. We used the EQ-VAS score as this might cover a broader underlying concept of overall health than the EQ-5D-5 L, which seems applicable for this population considering the heterogeneous and multidimensional character of BCa disease. If there is an actual change in HRQOL, we assumed that the EQ-VAS score shows this change. We constructed hypotheses about the change scores between T0 and T3 of the BCI and FACT-Bl-Cys, in comparison to the change scores of the EQ-VAS. We expected that patients with an improved or decreased EQ-VAS score (i.e. improvement or deterioration of ≥10 points), the BCI and FACT-Bl-Cys values also increased or declined, respectively. Previous studies in other patient populations reported a minimal important change (MIC) varying from 6.9 to 8.9 [45–48]. Based on these MIC values we assumed that an EQ-VAS change of 10 points might indicate actual change. Pre-specified hypotheses regarding the correlations and effect sizes between changes were that in patients with an improved or declined EQ-VAS score (i.e. plus or minus ≥10 points), we expected a) to find moderate correlations of ≥0.30 between change in EQ-VAS and the change in BCI and FACT-Bl-Cys values; b) to find a larger effect size, in comparison to patients that did not show a change in EQ-VAS (i.e. plus or minus ≤5 points). We analyzed the effect size between the changes by calculating Cohens d, using the formula: (T3_mean_-T0_mean_)/T0_SD_.

## Results

### Translation procedure

Conceptual rather than literal translation was leading to preserve the original meaning of each item. There was agreement between the forward and backward translations, and no cultural adaptions regarding translation were made. Based on the cognitive interviews, two items regarding the interpretation and meaning were discussed with the authors (JG, CM, CW) and FACIT representatives. First, we added one additional phrase “for men only” (in Dutch: “alleen voor mannen”) to the beginning of FACT-Bl-Cys item Bl5 “I am able to maintain an erection”, as this item is specifically aimed at men. According to FACIT scoring instructions Bl5 was not included in the scoring algorithm of the scale. Therefore, adapting this item could not influence the total score. Second, the BCI items where patients are asked about their “urine loss” could be interpreted according to their own specific situation. In case of a neobladder, stoma, or own bladder questions about “urine loss” could be answered as problems with catheterizing incontinence, emptying the stoma bag or leakage from the stoma bag, or urination, respectively.

### Patient population

In total 260 patients completed the baseline measure (T0), followed by 182 patients who completed the three-month measure post-operatively (T3). The T3 evaluation was completed at a mean of 106 days (SD:14 days). Sixty-two patients participated in the test–retest measurement (T3 retest). The T3 retest was completed at a mean of 14 days (SD:6 days) after T3. In Table 1 the characteristics of the study population are shown. Of the 260 patients who completed T0, the mean age was 67 years (SD:10 years), 207 (80%) were male, 177 (68%) were married, and 149 (57%) were retired. These characteristics were not notably different at T3. We did not find any major change scores between T0 and T3 for both measures, as presented in Table 2.

**Table 1:** Patient characteristics of the test and re-test groups

	Baseline (T0, pre-operatively)	3 months (T3, post-operatively)	Test-retest (T3 retest)^a^
Population size (n)	260	182	62
Age (years)			
Mean ± SD	67 ± 10	67 ± 9	67 ± 8
Median (min-max)	68 (35–88)	68 (38–82)	67 (40–82)
Gender, n (%)			
Women	53 (20%)	37 (20%)	14 (23%)
Men	207 (80%)	145 (80%)	48 (77%)
Type of surgery, n (%)			
RARC	142 (55%)	99 (54%)	32 (52%)
ORC	112 (43%)	78 (43%)	30 (48%)
LRC	6 (2%)	5 (3%)	0 (0%)
Diversion type, n (%)			
Own bladder	233 (90%)	0 (0%)	0 (0%)
Ileal conduit	11 (4%)	114 (63%)	45 (72%)
Neobladder	1 (0%)	33 (18%)	11 (18%)
Other	3 (1%)	31 (17%)	6 (10%)
Missing	12 (5%)	4 (2%)	0 (0%)
Preoperative chemotherapy (yes), n (%)	38 (15%)	28 (15%)	8 (13%)
Education, n (%)			
Elementary school or less	22 (8%)	17 (9%)	3 (5%)
Secondary education	94 (36%)	67 (37%)	20 (32%)
Postsecondary education	116 (45%)	87 (48%)	36 (58%)
Missing	28 (11%)	11 (6%)	3 (5%)
Living status, n (%)			
Married	177 (68%)	136 (75%)	51 (82%)
Other	83 (32%)	46 (25%)	11 (18%)
Employment status, n (%)			
Pension	149 (57%)	108 (59%)	36 (58%)
Salaried employment	61 (24%)	44 (25%)	19 (31%)
Other	23 (9%)	19 (10%)	4 (6%)
Missing	27 (10%)	11 (6%)	3 (5%)

^a^Two weeks after T3. *RARC* robot-assisted radical cystectomy, *ORC* open radical cystectomy, *LRC* laparoscopic-assisted radical cystectomy

**Table 2:** Change in scores between T0 (baseline) and T3 (90 days post-operative) of the measures

Domains	No. of items per domain	T0 Valid population (n)^a^	T0 Mean (SD)	T0 Median (min-max)	T3 Valid population (n) ^a^	T3 Mean (SD)	T3 Median (min-max)	T0-T3			
								Valid population (n)	Mean change (SD)	Min-max	SE
BCI											
Urinary domain	12	223	81.6 (18.2)	86.4 (7–100)	158	82.9 (17.6)	89.6 (25–100)	145	1.7 (21.5)	−55; 60	1.8
Function	4	242	84.8 (25.1)	100.0 (0–100)	144	74.8 (33.2)	100.0 (0–100)	137	−11.0 (39.6)	−100; 100	3.4
Bother	8	212	79.1 (20.7)	84.4 (11–100)	159	86.6 (13.0)	90.6 (47–100)	136	7.6 (20.4)	−41; 75	1.7
Bowel domain	10	239	86.6 (14.4)	91.7 (34–100)	170	81.4 (16.6)	86.7 (18–100)	160	−5.4 (18.9)	−79; 58	1.5
Function	4	234	86.3 (15.5)	91.8 (15–100)	163	83.8 (17.1)	87.5 (6–100)	152	−2.5 (20.3)	−79; 65	1.7
Bother	6	239	86.7 (17.0)	95.8 (25–100)	196	80.0 (18.5)	83.3 (21–100)	159	−6.5 (21.3)	−79; 63	1.7
Sexual domain	12	157	49.1 (19.6)	51.4 (9–85)	64	37.1 (17.7)	37.4 (3–75)	52	−15.6 (19.8)	− 62; 18	2.8
Function	7	148	33.0 (19.3)	31.3 (0–82)	52	22.7 (16.6)	25.0 (0–61)	42	−14.2 (18.6)	−52; 18	2.9
Bother	5	200	67.2 (30.7)	75.0 (0–100)	130	52.0 (31.0)	40.0 (0–100)	116	−13.5 (37.8)	−100; 100	3.5
FACT-Bl-Cys											
FACT-PWB	7	254	23.5 (4.6)	25.0 (7–28)	180	24.4 (3.6)	25.0 (8–28)	177	0.5 (4.5)	−16; 18	0.34
FACT-SWB	7	255	22.0 (4.4)	22.2 (0–28)	177	21.3 (4.5)	22.0 (0–28)	175	−1.0 (4.6)	−24; 22	0.35
FACT-EWB	6	253	17.8 (4.8)	19.0 (0–24)	173	20.4 (3.5)	21.0 (5–24)	167	2.8 (4.1)	−8; 23	0.32
FACT-FWB	7	257	17.7 (5.8)	18.0 (0–28)	175	19.0 (4.9)	20.0 (6–28)	172	1.1 (5.0)	−14; 20	0.38
Bl-Cys domain	15	257	40.7 (10.0)	41.8 (9–60)	175	43.9 (8.0)	45.0 (11–58)	172	2.5 (10.3)	−34; 28	0.79
FACT-Bl-Cys total score	42	247	121.8 (22.3)	124.0 (38–168)	169	129.5 (18.0)	132.0 (47–163)	161	5.7 (19.0)	−55; 71	1.5
EQ-5D											
EQ-5D-5 L	5	245	0.80 (0.1)	0.85 (0–1)	176	0.84 (0.13)	0.88 (0–1)	167	0.03 (0.17)	−1; 1	0.1
EQ-VAS	1	260	72.0 (20.5)	75.0 (0–100)	229	74.9 (19. 5)	80.0 (0–100)	229	−13.5 (36.9)	−96; 81	2.4

^a^The number of patients that completed enough items to calculate a total score, *PWB* Physical well-being domain, *SWB* Social/family well-being domain, *EWB* Emotional well-being domain, *FWB* Functional well-being domain, ^−^FACT-Bl-Cys total score is only calculated when all items have been completed

### Floor and ceiling effects

Seven out of nine BCI domains showed a ceiling effect (meaning few problems), while no floor effects were observed (see Table 3). Most missing values were reported in the BCI “sexual function” domain at T0; for 112 of 260 patients (43%) we could not calculate a domain score.

**Table 3:** Reliability properties of the BCI (T0, baseline) and FACT-Bl-Cys (T3, 90 days post-operative)

Domains	Population size (n)	Valid population (n)^a^	No. of items per domain	Minimal no. of missing items (%)^b^	Maximal no. of missing items (%)^c^	Mean (SD)	Median (min-max)	Floor effect	Ceiling effect	Internal consistency (Cronbach’s α)
T0 BCI										
Urinary domain	260	223	12	0 (66%)	12 (3%)	82 (18)	86 (7–100)	0 (0%)	41 (16%)	0.83
Function	260	241	4	0 (93%)	4 (4%)	85 (25)	100 (0–100)	5 (2%)	144 (55%)	0.86
Bother	260	212	8	0 (67%)	8 (4%)	79 (21)	84 (11–100)	0 (0%)	48 (19%)	0.82
Bowel domain	260	239	10	0 (88%)	10 (4%)	87 (15)	92 (34–100)	0 (0%)	40 (15%)	0.83
Function	260	234	4	0 (90%)	4 (4%)	86 (15)	92 (15–100)	0 (0%)	92 (35%)	0.70
Bother	260	239	6	0 (90%)	6 (4%)	87 (17)	96 (25–100)	0 (0%)	73 (35%)	0.77
Sexual domain	260	157	12	0 (42%)	12 (12%)	49 (20)	51 (9–85)	0 (0%)	0 (0%)	0.91
Function	260	148	7	0 (45%)	7 (12%)	33 (19)	31 (0–82)	5 (2%)	0 (0%)	0.90
Bother	260	200	5	0 (69%)	5 (15%)	67 (31)	75 (0–100)	5 (2%)	61 (24%)	0.85
T3 FACT-Bl-Cys										
FACT-PWB	182	180	7	0 (91%)	6 (1%)	24 (4)	25 (0–28)	0 (0%)	25 (14%)	0.80
FACT-SWB	182	177	7	0 (63%)	7 (2%)	21 (4)	22 (0–28)	2 (1%)	11 (6%)	0.81
FACT-EWB	182	173	6	0 (92%)	6 (4%)	20 (3)	21 (5–24)	0 (0%)	1 (1%)	0.77
FACT-FWB	182	175	7	0 (90%)	7 (4%)	19 (5)	20 (6–28)	1 (1%)	1 (1%)	0.85
Bl-Cys domain	182	175	15	0 (63%)	15 (4%)	44 (8)	45 (11–58)	0 (0%)	0 (0%)	0.77
FACT-Bl-Cys total^−^	182	169	42	–	–	129 (18)	132 (74–163)	0 (0%)	0 (0%)	0.90

^a^The number of patients that completed enough items to calculate a total score, ^b^Minimal number of missings per domain (i.e. 67% misses 0 items), ^c^Maximal number of missings per domain (i.e. 3% misses 12 items), *PWB* Physical well-being domain, *SWB* Social/family well-being domain, *EWB* Emotional well-being domain, *FWB* Functional well-being domain, ^−^FACT-Bl-Cys total score is only calculated when all items have been completed

Regarding the FACT-Bl-Cys, no floor or ceiling effects were detected (see Table 3). The FACT-EWB domain at T3 was missing for 9 out of 182 patients (5%). For 13 out of 182 patients (7%) the FACT-Bl-Cys total score could not be calculated since they did not complete all items.

### Validity

Face validity was considered to be good for both measures, as they appeared to address all relevant aspects of HRQOL in Dutch BCa adults treated with radical cystectomy.

Regarding content validity, interviewed patients expressed an overall positive opinion regarding relevance and comprehensiveness of the measures and their evaluative purposes. Nonetheless, one item of FACT-Bl-Cys (Bl1 “I have trouble controlling my urine”) raised concerns by some respondents, as patients with different bladder diversions might interpret this differently (e.g. stoma, neobladder of bladder replacement). We discussed these findings with FACIT representatives, and decided that patients could respond to this item in any way they feel appropriate.

The CFA was performed on the imputed datasets and the model fit was evaluated based on Comparative Fit Index (CFI), Tucker-Lewis index (TLI), Root Means Square Error of Approximation (RMSEA), and Standardized Root Mean Square Residual (SRMR). Considering structural validity for BCI, the CFA resulted in a moderate fit (see Table 4). The BCI in six factors showed a better fit (CFI:0.88, TLI:0.87, RMSEA:0.06, SRMR:0.07) in comparison to three factors (CFI:0.69, TLI:0.67, RMSEA:0.09, SRMR:0.09). Considering structural validity for FACT-Bl-Cys, the CFA also resulted in a moderate fit (see Table 4). For FACT-G in four factors, the CFI was 0.88, the TLI was 0.86, the RMSEA was 0.05, and the SRMR was 0.09. The Bl-Cys domain in three factors as proposed by Kim et al. [18] showed better fit (CFI:0.85, TLI:0.81, RMSEA:0.06, SRMR:0.09). None of the hypothesized factor structures for both PROMs fulfilled the CFI or TLI thresholds of ≥0.95.

**Table 4:** Structural validity properties of the BCI and FACT-Bl-Cys

	CFI	RMSEA	SRMR	TLI
T0 BCI				
Three-factor structure (Gilbert et al. [13])	0.69	0.09	0.09	0.67
1. Urinary domain (12 items)				
2. Bowel domain (10 items)				
3. Sexual domain (12 items)				
Six-factor structure (Hever et al. [16])	0.88	0.06	0.06	0.87
1. Urinary function (4 items)				
2. Urinary bother (8 items)				
3. Bowel function (4 items)				
4. Bowel bother (6 items)				
5. Sexual function (7 items)				
6. Sexual bother (5 items)				
T3 FACT-Bl-Cys				
FACT-G in four-factor structure (27 items)	0.88	0.05	0.09	0.86
1. PWB Physical well-being domain (7 items)				
2. SWB Social/family well-being domain (7 items)				
3. EWB Emotional well-being domain (6 items)				
4. FWB Functional well-being domain (7 items)				
Bl-Cys domain in three-factor structure (Anderson et al. [11])	0.75	0.07	0.11	0.70
1. C2 Losing weight; C3 Bowel control; C4 Diarrhea; C6 Good appetite; ITU6 Embarrassed my condition; ITU1 Comfortable discussing with friends (6 items)				
2. C7 Content with appearance; ITU3 Limit social interactions; ITU4 Limit physical activity; ITU5 Limit sexual activity (4 items)				
3. Bl1 Trouble controlling urine; ITU7 Condition wakes me up at night; C9 Caring for condition difficult; VC1 Satisfied with urinary condition; ITU2 I am afraid to be far from a toilet (5 items)				
Bl-Cys domain in three-factor structure (Kim et al. [18])	0.85	0.06	0.09	0.81
1. C2 Losing weight; C4 Diarrhea; Bl1 Trouble controlling urine; ITU7 Condition wakes me up at night; ITU6 Embarrassed my condition; C9 Caring for condition difficult; ITU2 I am afraid to be far from a toilet (7 items)				
2. C3 Bowel control; C6 Good appetite; C7 Content with appearance; ITU1 Comfortable discussing with friends; VC1 Satisfied with urinary condition (5 items)				
3. ITU3 Limit social interactions; ITU4 Limit physical activity; ITU5 Limit sexual activity (3 items)				

The CFA was performed using imputed data. *CFI* comparative fit index, *RMSEA* root mean square error of approximation; *SRMR* standardized root mean square residual, *TLI* Tucker-Lewis index, The criteria for unidimensionality include CFI ≥ 0.95, TLI ≥ 0.95, RMSEA≤0.06, and SRMR≤0.08

Considering hypotheses testing for BCI, 67% of prior hypotheses (8 of 12) were confirmed (see Table 5). Correlations among the three BCI domains were expected to be low, however we found a moderate correlation between the urinary domain and the bowel domain (r = 0.35). Although we expected moderate correlations when comparing the urinary domain with FACT-G, the sexual domain with EQ-5D-5 L, and the sexual domain with EQ-VAS, we instead found low correlations of r = 0.29, r = 0.24, r = 0.28, respectively. For FACT-Bl-Cys, 92% of all a priori hypotheses (12 of 13) were confirmed (see Table 5). As hypothesized for FACT-Bl-Cys, we found moderate correlations when comparing the Bl-Cys domain with EQ-5D-5 L (r = 0.60), FACT-Bl-Cys with EQ-5D-5 L (r = 0.70), and FACT-Bl-Cys with EQ-VAS (r = 0.58). All correlations between the five domains were moderate as expected, with the exception of one low correlation was observed (EWB-SWB r = 0.29).

**Table 5:** Construct validity properties of the BCI and FACT-Bl-Cys

Correlation expected	Hypotheses tested		Correlation coefficient (r)	Confirmed?
T0 BCI				
Low (< 0.30)	Correlations between 3 domains are low	Urinary – Bowel	0.35	No
		Urinary – Sexual	0.15	Yes
		Bowel – Sexual	0.25	Yes
Moderate (0.30–0.69)	Correlations between 3 domains and FACT-G are moderate	FACT-G – Urinary	0.29	No
		FACT-G – Bowel	0.39	Yes
		FACT-G – Sexual	0.36	Yes
	Correlations between 3 domains and EQ-5D-5 L are moderate	EQ-5D-5 L – Urinary	0.34	Yes
		EQ-5D-5 L – Bowel	0.38	Yes
		EQ-5D-5 L – Sexual	0.24	No
	Correlations between 3 domains and EQ-VAS are moderate	VAS – Urinary	0.37	Yes
		VAS – Bowel	0.32	Yes
		VAS – Sexual	0.28	No
T3 FACT-Bl-Cys				
Moderate (0.30–0.69)	Correlations between 5 domains are moderate	PWB – SWB	0.30	Yes
		PWB – EWB	0.51	Yes
		PWB – FWB	0.65	Yes
		PWB – Bl-Cys	0.63	Yes
		SWB – EWB	0.29	No
		SWB – FWB	0.41	Yes
		SWB – Bl-Cys	0.35	Yes
		EWB – FWB	0.59	Yes
		EWB – Bl-Cys	0.50	Yes
		FWB – Bl-Cys	0.64	Yes
	Correlation between Bl-Cys domain and EQ-5D-5 L is moderate	Bl-Cys domain – EQ-5D-5 L	0.60	Yes
	Correlation between total FACT-Bl-Cys and EQ-5D-5 L / EQ-VAS are moderate	FACT-Bl-Cys total score – EQ-5D-5 L	0.70	Yes
		FACT-Bl-Cys total score – VAS	0.58	Yes

### Reliability

Internal consistency (Cronbach’s α), test–retest reliability (ICC), and measurement error (SEM, SDC group, SDC individual) are presented in Tables 3 and 6.

**Table 6:** Test-retest properties of the BCI and FACT-Bl-Cys (T3 and T3 retest)

Domains	Valid population (n)	No. of items per domain	T3 Mean (SD)	T3 Median (min-max)	T3 retest Mean (SD)	T3 retest Median (min-max)	T3-T3 retest			T3 retest:^a^ ICC_agreement_ (95% CI)	Measurement error:^b^ SEM_agreement_	Measurement error:^c^ SDC_individual_	Measurement error:^d^ SDC_group_
							Mean change (SD)	95% CI	SE				
BCI													
Urinary domain	53	12	84 (17)	91 (32–100)	86 (15)	92 (31–100)	−2 (7)	−25; 15	0.9	0.90 (0.83; 0.91)	4.98	13.82	1.90
Function	43	4	76 (33)	100 (0–100)	83 (28)	100 (0–100)	−7 (16)	−57; 8	2.4	0.85 (0.71; 0.92)	12.16	33.72	5.14
Bother	52	8	88 (12)	91 (50–100)	88 (14)	92 (47–100)	0 (9)	−17; 36	1.2	0.77 (0.63; 0.86)	6.13	16.99	2.36
Bowel domain	61	10	84 (14)	88 (37–100)	86 (14)	90 (47–100)	−2 (10)	−26; 26	1.3	0.76 (0.63; 0.85)	7.05	19.54	2.50
Function	57	4	85 (17)	92 (36–100)	87 (14)	92 (42–100)	−2 (11)	−33; 29	1.5	0.74 (0.60; 0.84)	7.80	21.63	2.86
Bother	61	6	83 (16)	88 (33–100)	85 (16)	92 (38–100)	−2 (11)	−29; 26	1.4	0.76 (0.63; 0.85)	7.98	22.12	2.83
Sexual domain	19	12	40 (16)	42 (3–65)	38 (17)	35 (8–69)	2 (11)	−11; 26	2.5	0.78 (0.53; 0.91)	7.55	20.92	4.80
Function	14	7	26 (16)	29 (0–48)	29 (16)	29 (4–54)	−3 (5)	−14; 8	1.4	0.93 (0.79; 0.98)	4.17	11.56	3.09
Bother	42	5	62 (28)	60 (6–100)	55 (26)	50 (13–100)	7 (28)	−59; 80	4.3	0.47 (0.20; 0.67)	20.07	55.63	8.58
FACT-Bl-Cys													
FACT-PWB	58	7	25 (4)	26 (13–28)	25 (3)	26 (16–28)	0 (2)	−7; 8	0.3	0.74 (0.59; 0.83)	1.76	4.88	0.64
FACT-SWB	57	7	22 (4)	22 (0–28)	22 (3)	23 (13–28)	−1 (4)	− 19; 5	0.6	0.43 (0.19; 0.62)	3.01	8.34	1.10
FACT-EWB	54	6	21 (3)	21 (11–24)	21 (3)	22 (12–24)	0 (2)	−4; 7	0.3	0.72 (0.56; 0.83)	1.57	4.36	0.59
FACT-FWB	54	7	20 (5)	21 (6–28)	20 (5)	22 (9–28)	−1 (3)	−8; 8	0.5	0.78 (0.64; 0.86)	2.35	6.52	0.89
Bl-Cys domain	54	15	45 (7)	44 (24–57)	46 (8)	47 (20–59)	0 (4)	−10; 13	0.6	0.82 (0.71; 0.89)	3.14	8.69	1.18
EQ-5D-5 L													
5D-5 L	55	5	0.87 (0.1)	0.91 (0.49–0.95)	0.85 (0.1)	0.88 (0.54–0.95)	0 (0.1)	−0.1;0.2	0.01	0.82 (0.71; 0.89)	0.05	0.13	0.02
EQ-VAS	62	1	75 (22)	81 (0–100)	73 (26)	81 (0–100)	2 (30)	−85;87	4	0.23 (−0.26; 0.45)	21.21	58.79	7.47

^a^*ICC* Intraclass Correlation Coefficient (two-way random effects model, absolute agreement, single measures); ^b^*SEM* Standard Error of Measurement (√(σ^2^_o_ + σ^2^_residual_)); ^c^*SDC*_*individual*_ Smallest Detectable Change on individual level (=1.96*√ (2)*SEM); ^d^*SDC*_*group*_ Smallest Detectable Change on group level, i.e. the smallest difference in score that can be distinguished from measurement errors (=SDC_individual_/√(n))

For BCI, Cronbach’s α of the domains ranged from 0.70 (“bowel function”) to 0.91 (sexual domain), which indicates a good internal consistency. All ICCs were above 0.70, except for “sexual bother” which showed an ICC of 0.47. The SEM ranged from 4.17 (“sexual function”) to 20.07 (“sexual bother”), which resulted in a SDC individual score ranging from 11.56 to 55.63. The SDC group ranged from 1.90 (urinary domain) to 8.58 (“sexual bother”). Thus for the domain “sexual bother”, an average group difference in score ≥ 8.58 cannot be attributed to only measurement error, but actual change will have taken place.

For FACT-Bl-Cys, Cronbach’s α of the domains ranged from 0.77 (Bl-Cys) to 0.85 (FWB), which indicates a good internal consistency. All ICCs were above 0.70, except for SWB which showed an ICC of 0.43. The SEM ranged from 1.57 (EWB) to 3.14 (Bl-Cys domain), which resulted in a SDC individual score ranging from 4.36 to 8.69. Our SDC individual values show that, to determine a treatment effect a difference of at least 4.36 to 8.69 points between two scores from an individual patient is required to ensure that the difference is not attributed to measurement error. The SDC group ranged from 0.59 (EWB) to 1.18 (Bl-Cys). See Additional file 1 for separate variance components.

### Responsiveness

Our total study population did not show major changes between T0 and T3 for the BCI and FACT-Bl-Cys (see Table 2). When evaluating the occurrence of complications, it appeared that our dataset at the time contained too few registered complications to assess responsiveness, see Additional file 2. Only 42 patients had at least one registered complication (Clavien-Dindo classification grade 1 or higher). The number of patients that completed enough items to calculate a total score ranged from 0 to 27, depending on the domain.

Additionally, the change score between T0 and T3 of BCI and FACT-Bl-Cys in comparison to the change score of the EQ-VAS was studied. As presented in Additional file 3, mean change scores between T0 and T3 were calculated for patients who showed an improvement in the EQ-VAS score of ≥10 points (*n* = 55, mean change:25, SD:16) or a deterioration in this score of ≥10 points (*n* = 89, mean change:-50, SD:30).

For BCI, the number of patients with an improved EQ-VAS ranged from 17 to 47, depending on the domain. We hypothesized to find a larger effect size in the improved group compared to the no-change group, but only 1 of 9 hypotheses was confirmed (see Table 7). We only found a larger effect size for the urinary domain in the improved group, in comparison to the no-change group (Cohens d: 0.36 > 0.06). Regarding hypotheses on correlations, only 22% of all a priori hypotheses (2 of 9) were confirmed. We only found the expected moderate correlations for the change score of the bowel domain (r = 0.42) and the “bowel bother” domain (r = 0.39).

**Table 7:** Responsiveness properties of the BCI and FACT-Bl-Cys

Domains	Valid population (n) ^a^			Observed correlation changes ^f^			Hypotheses on correlations: confirmed? ^g^		Observed effect size ^e^			Hypotheses on effect sizes: confirmed? ^h^	
	EQ-VAS no change group ^b^	EQ-VAS improved group ^c^	EQ-VAS deteriorated group ^d^	EQ-VAS no change group ^b^	EQ-VAS improved group ^c^	EQ-VAS deteriorated group ^d^	EQ-VAS improved group ^c^	EQ-VAS deteriorated group ^d^	EQ-VAS no change group ^b^	EQ-VAS improved group ^c^	EQ-VAS deteriorated group ^d^	EQ-VAS improved group ^c^	EQ-VAS deteriorated group ^d^
EQ-VAS	68	55	89	1.00	1.00	1.00	NA.	NA.	0.00	1.32	−2.94	NA.	NA.
BCI													
Urinary domain	55	42	32	0.10	0.17	−0.12	No	No	0.06	0.36	−0.44	Yes	Yes
Function	51	43	29	0.00	−0.13	− 0.19	No	No	−0.45	− 0.44	− 0.56	No	Yes
Bother	52	38	31	0.04	0.29	−0.05	No	No	0.35	0.35	−0.05	No	No
Bowel domain	57	47	40	0.32	0.42	0.11	Yes	No	−0.90	−0.06	−0.44	No	No
Function	53	46	38	0.24	0.25	−0.18	No	No	−0.43	0.05	−0.36	No	No
Bother	57	47	39	0.29	0.39	0.18	Yes	No	−0.75	−0.11	−0.42	No	No
Sexual domain	15	20	10	−0.01	0.23	−0.14	No	No	−0.85	−0.50	−1.10	No	Yes
Function	14	17	12	0.01	0.25	−0.36	No	Yes	−0.68	−0.48	−1.25	No	No
Bother	43	34	28	0.06	0.04	0.28	No	No	−0.35	−0.29	−0.72	No	No
FACT-Bl-Cys													
FACT-PWB	66	52	42	0.54	0.51	0.44	Yes	Yes	0.00	0.80	−0.40	Yes	Yes
FACT-SWB	65	52	41	0.26	0.20	0.24	No	No	−0.50	−0.17	0.00	No	No
FACT-EWB	61	49	40	0.30	0.40	0.38	Yes	Yes	0.60	0.80	0.25	Yes	No
FACT-FWB	64	51	40	0.42	0.47	0.31	Yes	Yes	0.20	0.67	−0.40	Yes	Yes
Bl-Cys domain	64	50	41	0.13	0.44	0.42	Yes	Yes	0.22	0.55	−0.22	Yes	Yes
FACT-Bl-Cys total	59	45	40	0.39	0.62	0.53	Yes	Yes	0.17	0.75	−0.18	Yes	Yes

^a^ The number of patients that completed enough items to calculate a total score; ^b^ No change between T0 and T3 for patients in EQ-VAS score (plus or minus ≤5 points); ^c^ Change between T0 and T3 for patients with an improved EQ-VAS score of ≥10 points; ^d^ Change between T0 and T3 for patients with a deteriorated EQ-VAS score of ≥10 points; ^e^ Formula assessed for Effect Size (Cohens d): ChangeT3T0_Mean_/T0_SD_; ^f^ Spearman correlation (r) between change score EQ-VAS and change score BCI and FACT-Bl-Cys (T0 and T3);

^g^ Hypotheses correlations = in patients with an improved or declined EQ-VAS score (≥10 points), we hypothesized to find moderate correlations of ≥0.30 between change in EQ-VAS and the change in BCI and FACT-Bl-Cys values;

^h^ Hypotheses effect sizes = in patients with an improved or declined EQ-VAS score (≥10 points), we hypothesized to find a larger effect size, in comparison to patients that did not show a change in EQ-VAS (≤5 points)

For BCI, the number of patients with a deteriorated EQ-VAS ranged from 10 to 40, depending on the domain. Regarding hypotheses on effect sizes for the deterioration group, only 3 out of 9 hypotheses were confirmed. We found larger effect sizes in the urinary domain (Cohens d: 0.44 > 0.06), “urinary function” (Cohens d: − 0.56 > − 0.45), and the sexual domain (Cohens d: − 1.10 > − 0.85). Regarding hypotheses on correlations, only 11% of all a priori hypotheses (1 of 9) were confirmed. We only found a moderate correlation for the change score of the “sexual function” domain.

For FACT-Bl-Cys, the number of patients with an improved EQ-VAS ranged from 45 to 52, depending on the domain. For both effect sizes and correlations, 83% of all a priori hypotheses (5 of 6) were confirmed. Only for FACT-SWB we found a smaller effect size (Cohens d: − 0.17 < − 0.50) and a low correlation (r = 0.20).

For FACT-Bl-Cys, the number of patients with a deteriorated EQ-VAS ranged from 40 to 42, depending on the domain. Regarding hypotheses on effect sizes for the deterioration group, 67% of all a priori hypotheses (4 of 6) were confirmed (see Table 7). We found lower effect sizes in the FACT-SWB (Cohens d: 0.00 < -0.50) and FACT-EWB (Cohens d: 0.25 < 0.60). Regarding hypotheses on correlations, 83% of all a priori hypotheses (5 of 6) were confirmed. We only found a low correlation for the change in FACT-SWB (r = 0.24), which was contrary to our expectations. Overall, these results indicate that the FACT-Bl-Cys total score seems responsive to change in generic HRQOL.

## Discussion

The aim of this study was to translate two disease-specific measures (BCI and FACT-Bl-Cys) into Dutch and to evaluate their measurement properties according to the COSMIN criteria. Both measures were found to be reliable and have good content validity, but limited structural validity. This study showed that the Dutch version of the BCI was not very responsive to changes in generic HRQOL, and showed a ceiling effect. In the Dutch version of the FACT-Bl-Cys no floor or ceiling effects were observed and the FACT-Bl-Cys total score was responsive to changes in generic HRQOL.

The structural validity of the BCI was assessed as moderate, as the hypothesized domains based on the original validation [13] and translational study [16] could partially be confirmed. For BCI, we found a better fit for a six-factor than a three-factor structure, which could indicate that BCI distinguishes function and bother. The structural validity of the FACT-Bl-Cys was assessed as moderate as the majority of hypothesized domains could not be confirmed. This limited fit could be caused by the heterogeneous characteristics of BCa disease, as many different topics are covered in the domains. The FACT-Bl-Cys domains contain a wide range of complaints that do not necessarily relate to each other. For instance, the Bl-Cys domain contains items related to body image, physical functioning, satisfaction, and socials aspects. Future exploration of whether there is a more adequate component solution is recommended for further research.

Although experts debate on the minimum number of patients needed for a factor analysis (ranging from 100 to 300 patients) [49–54], COSMIN advises to use a sample size of 7 patients per item, with a minimum number of 100 patients [38]. Though we handled the missing data with multiple imputation in order to perform the CFA with an adequate sample size, when interpreting the results it should be considered that due to multiple imputation, in which missing values are imputed with the assumption that the distribution of the missing values is similar to the observed data (i.e. predictive mean matching approach), indices might be overfitted.

Regarding BCI at T0, high ceiling effects were observed for both urinary and bowel domains. The maximum scores indicate good function and no bother, which might be when patients are recently diagnosed and have minor symptoms (e.g. painless hematuria). Similar findings are reported in previous studies [13, 16, 17].

Regarding FACT-Bl-Cys, there were uncertainties concerning the calculation of total scores, as we excluded the items BL4 ‘I am interested in sexual activity’ and BL5 ‘I am able to have and maintain an erection’, according to FACIT instructions. These items were also not included in the CFA. Previously published studies each handled these items differently [11, 12, 18, 19].

In comparison to previous BCI [13, 16, 17] and FACT-Bl-Cys [11, 18, 19] studies, we found similar internal consistency values for all domains. Reliability of both measures in terms of test-retest scores was good. To our knowledge, the SEM and SDC have not yet been assessed before for both measures. It should be noticed that our design methodologically differs from previous conducted studies. Previous FACT-Bl-Cys studies [11, 12, 18, 19] assessed test-retest reliability using an interval of 4 weeks, while COSMIN advises to use an interval of 2 weeks [38]. Additionally, FACT-Bl-Cys was completed at different moments (e.g. 7 months to 3 years post-operatively) [11, 12, 18, 19]. Anderson et al. did not show an ICC, but reported a test-retest Spearman correlation of 0.89 for the Bl-Cys domain [11]. Three other studies reported an ICC of 0.79 [12], 0.75 [19], and 0.82 [18] for the Bl-Cys domain, which is similar to our ICC of 0.82.

Regarding construct validity of the BCI, our results confirm the suggestion that the BCI captures additional information which is not covered by generic instruments. Schmidt et al. found similar results as they reported low to moderate correlations (range: 0.16–0.48) between BCI and the SF-36 [17]. Regarding construct validity of the FACT-Bl-Cys, only Kim et al. correlated FACT-Bl-Cys with a generic measure [18]. They found moderate correlations (range: 0.29–0.69) between FACT-Bl-Cys and the SF-36 [18]. These results are comparable to our moderate correlations between FACT-Bl-Cys and the generic measures. In contrast to our results, three previous studies showed a low correlation (range: 0.16–0.24) between SWB and Bl-Cys domain [12, 18, 19]. Cookson et al. suggested that this low correlation reflects the predominant focus on physical/functional aspects of HRQOL in the FACT-Bl-Cys [12]. We found moderate correlations between all four domains (PWB, SWB, EWB, FWB) and the Bl-Cys domain, which indicates an equal share in HRQOL.

Only one BCI study mentioned responsiveness [17]. In 2014, Schmidt et al. reported an improvement 12 months after treatment for the urinary domain (Cohens d: 0.38) and “urinary bother” (Cohens d: 0.53). Based on these findings, the authors concluded that the BCI is responsive [17]. In 2018, a systematic review performed by Mason et al., however, rated the BCI responsiveness as unknown because Schmidt et al. only calculated effect sizes and did not construct hypotheses a priori which is not conform COSMIN recommendations [4].

We found one study that reported on longitudinal changes of FACT-Bl-Cys [11]. Anderson et al. evaluated change in 190 patients who underwent a radical cystectomy (pre-operative and 12 months post-operative) [11]. Authors did not calculate an effect size or correlations regarding responsiveness according to COSMIN, but they reported that patients with an ileal conduit diversion had an average 5 points higher score at 12 months, than those with an orthotopic neobladder diversion.

### Strengths and limitations

As far as we are aware, this is the first study determining the measurement properties of BCI and FACT-Bl-Cys, according to COSMIN guidelines, in a large study population. COSMIN enhances clarity and stimulates uniform usage of terminology, enables researchers to evaluate each measurement property individually, and improves comparability with other studies.

This study also has some limitations. First, our study population comprised only patients treated with radical cystectomy. Therefore, we were unable to determine whether both measures will be valid and reliable in a more generic BCa population. Second, due to our study design test-retest and measurement error had to be determined at 3 months (T3, post-operatively) rather than at baseline (T0, pre-operatively). For FACT-Bl-Cys this is an adequate moment, but for BCI this might result in a bias since this measure is originally intended for BCa patients in general. Third, to explore responsiveness we studied the correlation and effect size of change score of both measures with the change score of the EQ-VAS between T0 and T3. Although we expected that radical cystectomy impacts HRQOL, the BCI and FACT-Bl-Cys did not detect large changes between T0 and T3. Two previous studies did also not find major changes between T0 and T3 for FACT-Bl-Cys [55, 56]. This small change could indicate that radical cystectomy does not impact HRQOL largely, but it is also possible that the BCI and FACT-Bl-Cys do not adequately detect the actual impact and change over time. Particularly since we did find a large change in EQ-VAS scores: the population with an improved EQ-VAS score (*n* = 55) showed a mean increase of 25 points, and the population with a deteriorated EQ-VAS score (*n* = 89) showed a mean decrease of 50 points. Previous studies in other patient populations reported MIC values varying from 6.9 to 8.9 [45–48], but comparability between these different populations should be considered. The FACT-Bl-Cys appeared to be responsive to changes in generic HRQOL, though limited structural validity must be considered as this could negatively impact the responsiveness of specific domains. The possibility remains that in some cases responsiveness was not detected, because there was no meaningful change. In our study population we found too few registered complications to assess responsiveness based on the occurrence of complications. This study was a first exploration of responsiveness of both measures, further research is warranted to evaluate whether the BCI and FACT-Bl-Cys are responsive to objective measures of change (e.g. occurrence of complications).

### Clinical implications

Based on this study, both measures seem suitable for cross-sectional use in clinical practice and for research purposes. Consulted urologists mentioned that all topics of both measures are usually discussed with patients in clinical practice, and usage of measures could be valuable for clinicians in order to provide more personalized care. Additionally, urologists noticed that the measures seem quite long for daily practice which limits the suitability and feasibility, but removing or combining items influences validity.

When using the BCI, it should be considered that the largest proportion of missing values was found in the sexual domain, which is also noticed by two previous studies [16, 17]. During cognitive interviews, some patients mentioned that the sexual domain items were clear but ‘irrelevant’ as they had no sexual life. Thus a ‘never’ response or a missing value could indicate no sexual bother or function, or the lack of activity. Therefore, it is questionable whether BCI is suitable to measure “sexual function” and “sexual bother”. Patients also indicated that BCI items about “urine loss” and FACT-Bl-Cys items about “controlling my urine” could be interpreted in different ways, as patients might have different bladder diversions (e.g. stoma, neobladder of bladder replacement). We recommend that future users of the measures provide an instruction which states that patients should answer these items according to their own specific situation.

## Conclusion

The Dutch versions of the BCI and FACT-Bl-Cys were shown to be reliable and have good content validity. Structural validity was limited for both measures. Only the FACT-Bl-Cys total score was responsive to changes in generic HRQOL. Despite some limitations, both measures are suitable for cross-sectional use in clinical practice and for research purposes. Exploration of a more adequate component solution and whether both PROMs are responsive when objective measures (e.g. occurrence of complications) are used as an indicator of change, needs to be further evaluated in future research.

## Additional files

Additional file 1: Variance components of the measures. (DOCX 22 kb)

Additional file 2: Change in scores between T0 (baseline) and T3 (90 days post-operative) of the measures in relation to registered complications. (DOCX 44 kb)

Additional file 3: Change in scores between T0 (baseline) and T3 (90 days post-operative) of the measures in relation responsiveness properties. (DOCX 26 kb)

## Data Availability

When the RACE study is completed, data will be made available in accordance with ZonMw regulations.

## Abbreviations

BCa: Bladder Cancer
BCI: Bladder Cancer Index
CFA: Confirmatory Factor Analyses
CFI: Comparative Fit Index
COSMIN: COnsensus-based Standards for the selection of health Measurement Instruments
EFA: Exploratory Factor Analyses
EQ-5D-5 L: EuroQol-5 Dimensions-5 Levels
EQ-VAS: EuroQol Visual Analogue Scale
ES: Effect size
EWB: Emotional well-being domain
FACIT: Functional Assessment of Chronic Illness Therapy
FACT-Bl-Cys: Functional Assessment of Cancer Therapy–Bladder cancer Cystectomy
FWB: Functional well-being domain
HRQOL: Health-related quality of life
ICC: Intraclass Correlation Coefficient
LRC: Laparoscopic-assisted Radical Cystectomy
MCAR: Missing Completely At Random
MIC: Minimal Important Change
NTR: Nederlands Trial Register (Dutch Trial Registry)
ORC: Open Radical Cystectomy
PRO: Patient Reported Outcomes
PWB: Physical well-being domain
QALY: Quality Adjusted Life Years
RACE: Radical Cystectomy Evaluation
RARC: Robot-assisted Radical Cystectomy
RMSEA: Root Means Square Error of Approximation
SDC: Smallest Detectable Change
SEM: Standard Error of Measurement
SRMR: Standardized Root Mean Square Residual
SWB: Social/family well-being domain
VAS: Visual Analogue Scale
