# Supplementary material for: Translation and validation of two disease-specific patient-reported outcome measures (Bladder Cancer Index and FACT-Bl-Cys) in Dutch bladder cancer patients
Source: J Patient Rep Outcomes. 2019 Sep 14;3:62. doi: 10.1186/s41687-019-0149-7 (PMC6745039; doi:10.1186/s41687-019-0149-7)
Supplement: Supplementary file 1 — Variance components of the measures. (DOCX 22 kb) [file 41687_2019_149_MOESM1_ESM.docx]

**Additional file 1**

Variance components of the measures

Assessed formulas: ICC _agreement_ = σ_p_^2^ / (σ_p_^2^ + σ_o_^2^ + σ_err_^2^)

SEM _agreement_ = √/ (σ_o_^2^ + σ_err_^2^)

Methods: restricted maximum likelihood estimation

| **Domains** | **Valid population (n)** | **Var**  **(patients, σ_p_^2^)** | **Var**  **(observations,σ_o_^2^)** | **Var**  **(error, σ_err_^2^)** | **ICC_agreement_** | **ICC_agreement_**  **95% CI** | **SEM_agreement_** |
| --- | --- | --- | --- | --- | --- | --- | --- |
| **BCI** | | | | | | | |
| Urinary-summary | 53 | 227.82 | 1.92 | 22.93 | 0.90 | 0.83; 0.91 | 4.98 |
| Function | 43 | 821.98 | 20.20 | 127.77 | 0.85 | 0.71; 0.92 | 12.16 |
| Bother | 52 | 127.84 | 0.00 | 37.55 | 0.77 | 0.63; 0.86 | 6.13 |
| Bowel-summary | 61 | 157.07 | 1.47 | 48.25 | 0.76 | 0.63; 0.85 | 7.05 |
| Function | 57 | 176.60 | 1.04 | 59.84 | 0.74 | 0.60; 0.84 | 7.80 |
| Bother | 61 | 200.62 | 1.95 | 61.72 | 0.76 | 0.63; 0.85 | 7.98 |
| Sexual-summary | 19 | 206.65 | 0.00 | 56.95 | 0.78 | 0.53; 0.91 | 7.55 |
| Function | 14 | 241.69 | 2.89 | 14.49 | 0.93 | 0.79; 0.98 | 4.17 |
| Bother | 42 | 356.39 | 17.63 | 385.16 | 0.47 | 0.20; 0.67 | 20.07 |
| **FACT-Bl-Cys** | | | | | | | |
| FACT-PWB | 58 | 8.57 | 0.03 | 3.07 | 0.73 | 0.59; 0.83 | 1.76 |
| FACT-SWB | 57 | 6.73 | 0.00 | 9.05 | 0.43 | 0.19; 0.62 | 3.01 |
| FACT-EWB | 54 | 6.35 | 0.00 | 2.48 | 0.72 | 0.56; 0.83 | 1.57 |
| FACT-FWB | 54 | 19.07 | 0.10 | 5.43 | 0.78 | 0.64; 0.86 | 2.35 |
| Bl-Cys domain | 54 | 44.61 | 0.00 | 9.83 | 0.82 | 0.71; 0.89 | 3.14 |
| **EQ-5D-5L** | | | | | | | |
| ED-5L | 55 | 0.01 | 0.00 | 0.00 | 0.83 | 0.71; 0.89 | 0.05 |
| EQ-VAS | 62 | 133.94 | 0.00 | 449.86 | 0.23 | -0.26; 0.45 | 21.21 |
