# Supplementary material for: Translation and validation of two disease-specific patient-reported outcome measures (Bladder Cancer Index and FACT-Bl-Cys) in Dutch bladder cancer patients
Source: J Patient Rep Outcomes. 2019 Sep 14;3:62. doi: 10.1186/s41687-019-0149-7 (PMC6745039; doi:10.1186/s41687-019-0149-7)
Supplement: Supplementary file 2 — Change in scores between T0 (baseline) and T3 (90 days post-operative) of the measures in relation to registered complications. (DOCX 44 kb) [file 41687_2019_149_MOESM2_ESM.docx]

**Additional file 2**

Change in scores between T0 (baseline) and T3 (90 days post-operative ) of the BCI and FACT-Bl-Cys in patients who had **no** **complication** (n=218, registered 30-11-2018).

| **Domains** | **No. of items per domain** | **T0 Valid population**  **(n)** | **T0**  **Mean (SD)** | **T0**  **Median  (min-max)** | **T3 Valid population**  **(n)** | **T3**  **Mean (SD)** | **T3**  **Median  (min-max)** | **T0-T3** | | | |
| --- | --- | --- | --- | --- | --- | --- | --- | --- | --- | --- | --- |
|  |  |  |  |  |  |  |  | **Valid population (n)** | **Mean change (SD)** | **Median change**  **(min;max)** | **Effect Size**  **(Cohens D)** |
| **BCI** | | | | | | | | | | | |
| Urinary-summary | 12 | 186 | 82.5 (17.8) | 86.6 (7-100) | 134 | 84.5 (16.0) | 90.0 (31-100) | 123 | 2.1 (20.0) | 0.6 (-53;60) | 0.12 |
| Function | 4 | 203 | 86.1 (24) | 100 (0-100) | 125 | 76.7 (31.3) | 100.0 (0-100) | 120 | -11.3 (39.6) | 0.0 (-100;100) | -0.46 |
| Bother | 8 | 175 | 79.7 (20.6) | 84.4 (11-100) | 135 | 87.4 (12.4) | 90.6 (47-100) | 115 | 7.5 (19.6) | 1.3 (-31;75) | 0.37 |
| Bowel-summary | 10 | 200 | 86.9 (13.7) | 91.7 (47-100) | 144 | 81.5 (16.8) | 86.7 (18-100) | 135 | -5.8 (19.1) | -3.3 (-79;52) | -0.42 |
| Function | 4 | 195 | 86.6 (15.0) | 91.8 (15-100) | 138 | 83.8 (17.4) | 87.5 (6-100) | 128 | -2.7 (20.8) | 0.0 (-79;65) | -0.18 |
| Bother | 6 | 200 | 87.1 (15.9) | 93.8 (25-100) | 143 | 80.3 (18.8) | 87.5 (21-100) | 134 | -6.8 (21.5) | -4.2 (-79;50) | -0.43 |
| Sexual-summary | 12 | 127 | 49.5 (19.2) | 52.5 (9-85) | 58 | 37.4 (17.9) | 39.1 (3-75) | 47 | -14.8 (18.6) | -11.2 (-62;18) | -0.77 |
| Function | 7 | 122 | 33.4 (18.5) | 32.6 (0-82) | 44 | 23.7 (16.7) | 27.0 (0-61) | 36 | -13.9 (16.5) | -14.5 (-52;18) | -0.75 |
| Bother | 5 | 166 | 67.0 (30.6) | 75.0 (0-100) | 144 | 53.1 (30.5) | 44.4 (0-100) | 103 | -11.9 (36.3) | -15.0 (-95;100) | -0.39 |
| **FACT-Bl-Cys** | | | | | | | | | | | |
| FACT-PWB | 7 | 212 | 23.6 (4.5) | 25.0 (7-28) | 153 | 24.6 (3.5) | 25.0 (8-28) | 150 | 0.6 (4.4) | 0.0 (-16;18) | 0.13 |
| FACT-SWB | 7 | 213 | 22.0 (4.4) | 22.2 (0-28) | 150 | 21.5 (4.5) | 22.0 (0-28) | 148 | -0.8 (4.7) | -1.0 (-24;22) | -0.18 |
| FACT-EWB | 6 | 211 | 17.7 (4.9) | 19.0 (0-24) | 148 | 20.4 (3.6) | 21.0 (5-24) | 142 | 2.9 (4.0) | 2.0 (-8;23) | 0.59 |
| FACT-FWB | 7 | 215 | 17.9 (5.8) | 19.0 (0-28) | 149 | 19.4 (4.9) | 20.0 96-28) | 146 | 1.3 (5.1) | 0.5 (-14;20) | 0.22 |
| Bl-Cys domain | 15 | 215 | 40.9 (9.8) | 41.8 (11-60) | 149 | 44.3 (7.9) | 45.0 (11-58) | 146 | 2.7 (9.8) | 2.0 (-33;28) | 0.28 |
| **EQ-5D** | | | | | | | | | | | |
| EQ-5D-5L | 5 | 205 | 0.80 (0.18) | 0.85 (0-1) | 150 | 0.85 (0.12) | 0.89 (0-1) | 143 | 0.04 (0.15) | 0.00 (0-1) | 0.22 |
| EQ-VAS | 1 | 218 | 71.4 (21.2) | 75.0 (0-100) | 190 | 62.0 (34.4) | 76.0 (0-100) | 190 | -10.6 (37.0) | 0.0 (-96;81) | -0.50 |

Change in scores between T0 (baseline) and T3 (90 days post-operative ) of the BCI and FACT-Bl-Cys in patients who had **at least one complication Grade 1-5**

(n=42, registered 30-11-2018).

| **Domains** | **No. of items per domain** | **T0 Valid population**  **(n)** | **T0**  **Mean (SD)** | **T0**  **Median  (min-max)** | **T3 Valid population**  **(n)** | **T3**  **Mean (SD)** | **T3**  **Median  (min-max)** | **T0-T3** | | | |
| --- | --- | --- | --- | --- | --- | --- | --- | --- | --- | --- | --- |
|  |  |  |  |  |  |  |  | **Valid population (n)** | **Mean change (SD)** | **Median change**  **(min;max)** | **Effect Size**  **(Cohens D)** |
| **BCI** | | | | | | | | | | | |
| Urinary-summary | 12 | 37 | 77.3 (19.4) | 79.9 (33-100) | 24 | 74.0 (23.3) | 79.4 (25-100) | 22 | -0.60 (28.7) | -0.2 (-55;48) | -0.03 |
| Function | 4 | 39 | 78.2 (29.6) | 91.8 (0-100) | 19 | 62.3 (42.3) | 91.8 (0-100) | 17 | -9.3 (40.1) | -8.3 (-83;67) | -0.31 |
| Bother | 8 | 37 | 76.3 (21.6) | 81.3 (25-100) | 24 | 82.1 (15.7) | 85.9 (50-100) | 21 | 8.2 (24.6) | 0.0 (-41;63) | 0.38 |
| Bowel-summary | 10 | 39 | 84.6 (19.6) | 82.5 (34-100) | 26 | 80.4 (15.2) | 85.9 (42-100) | 25 | -3.2 (17.7) | -5.0 (-36;58) | -0.16 |
| Function | 4 | 39 | 84.5 (17.7) | 91.8 (33-100) | 25 | 83.3 (15.5) | 93.8 (84-100) | 24 | -1.0 (18.0) | 0.0 (-33;52) | -0.06 |
| Bother | 6 | 39 | 84.7 (22.2) | 95.8 (29-100) | 26 | 78.8 (16.7) | 83.3 (38-100) | 25 | -4.7 (20.8) | -4.2 (-42;63) | -0.21 |
| Sexual-summary | 12 | 30 | 47.5 (21.5) | 49.2 (14-85) | 6 | 34.3 (17.1) | 31.6 (10-62) | 5 | -23.9 (30.4) | -31.9 (-54;18) | -1.11 |
| Function | 7 | 26 | 30.7 (23.0) | 27.4 (0-79) | 8 | 17.1 (15.8) | 18.8 (0-45) | 6 | -16.1 (30.5) | 16.9 (-49;17) | -0.70 |
| Bother | 5 | 34 | 68.2 (31.3) | 75.0 (0-100) | 16 | 44.5 (34.8) | 36.3 (0-100) | 13 | -26.0 (48.0) | -45.0 (-100;65) | -0.83 |
| **FACT-Bl-Cys** | | | | | | | | | | | |
| FACT-PWB | 7 | 42 | 23.2 (4.9) | 26.0 (9-28) | 27 | 23.4 (3.8) | 24.0 (12-28) | 27 | 0.0 (5.3) | -1.0 (-13;15) | 0.00 |
| FACT-SWB | 7 | 42 | 21.9 (4.4) | 22.5 (7-28) | 27 | 20.0 (4.4) | 21.0 (6-28) | 27 | -1.8 (4.0) | -1.0 (-8-9) | -0.41 |
| FACT-EWB | 6 | 42 | 18.7 (4.1) | 19.0 (3-24) | 25 | 20.4 (2.4) | 20.0 (14-24) | 25 | 2.3 (4.4) | 2.0 (-4;16) | 0.56 |
| FACT-FWB | 7 | 42 | 17.1 (5.6) | 17.0 (5-28) | 26 | 16.9 (4.7) | 17.5 (7-26) | 26 | 0.2 (3.8) | 0.0 (-7;7) | 0.04 |
| Bl-Cys domain | 15 | 42 | 39.7 (10.9) | 41.3 (9-60) | 26 | 41.5 (8.3) | 44.0 (19-60) | 26 | 1.3 (12.9) | 3.8 (-34;26) | 0.12 |
| **EQ-5D** |  |  |  |  |  |  |  |  |  |  |  |
| EQ-5D-5L | 5 | 40 | 0.81 (0.17) | 0.85 (0-1) | 26 | 0.79 (0.14) | 0.82 (0-1) | 24 | -0.04 (0.23) | 0.00 (-1;1) | -0.24 |
| EQ-VAS | 1 | 42 | 75.0 (16.2) | 80.5 (40-100) | 39 | 47.4 (35.5) | 68.0 (0-96) | 39 | -27.8 (33.3) | -15.0 (-92;31) | -1.72 |

Change in scores between T0 (baseline) and T3 (90 days post-operative ) of the BCI and FACT-Bl-Cys in patients who had **at least one minor complication Grade 1-2**

(n=33, registered 30-11-2018).

| **Domains** | **No. of items per domain** | **T0 Valid population**  **(n)** | **T0**  **Mean (SD)** | **T0**  **Median  (min-max)** | **T3 Valid population**  **(n)** | **T3**  **Mean (SD)** | **T3**  **Median  (min-max)** | **T0-T3** | | | |
| --- | --- | --- | --- | --- | --- | --- | --- | --- | --- | --- | --- |
|  |  |  |  |  |  |  |  | **Valid population (n)** | **Mean change (SD)** | **Median change**  **(min;max)** | **Effect Size**  **(Cohens D)** |
| **BCI** | | | | | | | | | | | |
| Urinary-summary | 12 | 29 | 78.6 (18.4) | 83.4 (43-100) | 20 | 73.3 (24.6) | 83.0 (25-100) | 18 | -3.4 (30.8) | -3.5 (-55;48) | -0.18 |
| Function | 4 | 30 | 83.7 (24.4) | 100.0 (17-100) | 16 | 64.6 (42.1) | 91.8 (0-100) | 14 | -10.1 (43.8) | -4.1 (-83;67) | -0.41 |
| Bother | 8 | 29 | 76.3 (22.2) | 81.3 (25-100) | 19 | 81.1 (16.6) | 84.4 (50-100) | 16 | 7.2 (25.6) | 0.0 (-41;63) | 0.32 |
| Bowel-summary | 10 | 30 | 86.3 (17.6) | 94.2 (39-100) | 21 | 80.6 (15.6) | 86.7 (42-100) | 20 | -4.2 (10.6) | -5.9 (-21;16) | -0.24 |
| Function | 4 | 30 | 86.2 (15.5) | 93.8 (42-100) | 21 | 82.6 (16.0) | 93.8 (48-100) | 20 | -2.3 (13.7) | 0.0 (-27;33) | -0.15 |
| Bother | 6 | 30 | 86.4 (20.4) | 95.8 (29-100) | 21 | 79.4 (17.2) | 83.3 (38-100) | 20 | -5.4 (13.0) | -4.2 (-29;21) | -0.26 |
| Sexual-summary | 12 | 23 | 47.8 (23.6) | 48.3 (14-85) | 6 | 34.3 (17.1) | 31.6 (10-62) | 5 | -23.9 (30.4) | -31.9 (-54;18) | -1.01 |
| Function | 7 | 21 | 31.5 (23.7) | 25.0 (0-79) | 7 | 17.2 (17.1) | 20.8 (0-45) | 5 | -22.6 (29.0) | -37.5 (-49;14) | -0.95 |
| Bother | 5 | 25 | 67.2 (33.0) | 70.0 (0-100) | 14 | 42.3 (33.3) | 42.3 (0-100) | 11 | -32.0 (42.9) | -45.0 (-100;60) | -0.97 |
| **FACT-Bl-Cys** | | | | | | | | | | | |
| FACT-PWB | 7 | 33 | 23.3 (5.1) | 26.0 (9-28) | 22 | 23.0 (4.1) | 24.0 (12-28) | 22 | -0.4 (5.4) | -1.0 (-13;15) | -0.08 |
| FACT-SWB | 7 | 33 | 22.7 (3.8) | 23.3 (12-28) | 22 | 21.3 (3.2) | 22.0 (14-28) | 22 | -1.8 (4.3) | -1.5 (-8;9) | -0.41 |
| FACT-EWB | 6 | 33 | 18.3 (4.5) | 19.0 (3-24) | 21 | 20.2 (2.5) | 20.0 (14-24) | 21 | 2.4 (4.6) | 1.0 (-3;16) | 0.53 |
| FACT-FWB | 7 | 33 | 17.3 (5.5) | 17.0 (5-28) | 21 | 17.5 (4.8) | 19.0 (26-26) | 21 | 0.0 (4.1) | -1.0 (-7;7) | 0.00 |
| Bl-Cys domain | 15 | 33 | 40.3 (11.4) | 42.0 (9-60) | 21 | 41.2 (9.2) | 44.0 (19-53) | 21 | 0.2 (13.6) | 3.5 (-34; 26) | 0.02 |
| **EQ-5D** | | | | | | | | | | | |
| EQ-5D-5L | 5 | 31 | 0.80 (0.19) | 0.85 (0-1) | 21 | 0.78 (0.15) | 0.81 (0-1) | 19 | -0.05 (0.26) | -0.03 (-1;1) | -0.26 |
| EQ-VAS | 1 | 33 | 76.0 (17.1) | 81.0 (40-100) | 31 | 50.9 (34.9) | 71.0 (0-96) | 31 | -25.8 (34.3) | -11.0 (-92;31) | -1.51 |

Change in scores between T0 (baseline) and T3 (90 days post-operative ) of the BCI and FACT-Bl-Cys in patients who had **at least one major complication Grade 3-5**

(n=9, registered 30-11-2018).

| **Domains** | **No. of items per domain** | **T0 Valid population**  **(n)** | **T0**  **Mean (SD)** | **T0**  **Median  (min-max)** | **T3 Valid population**  **(n)** | **T3**  **Mean (SD)** | **T3**  **Median  (min-max)** | **T0-T3** | | | |
| --- | --- | --- | --- | --- | --- | --- | --- | --- | --- | --- | --- |
|  |  |  |  |  |  |  |  | **Valid population (n)** | **Mean change (SD)** | **Median change**  **(min;max)** | **Effect Size**  **(Cohens D)** |
| **BCI** | | | | | | | | | | | |
| Urinary-summary | 12 | 8 | 72.5 (23.6) | 77.1 (33-100) | 4 | 77.2 (17.5) | 75.2 (58-100) | 4 | 12.1 (11.1) | 11.6 (0-26) | 0.51 |
| Function | 4 | 9 | 60.2 (39.1) | 66.8 (0-100) | 3 | 50.0 (50.0) | 50.0 (0-100) | 3 | -5.6 (19.1) | -16.5 (-17;17) | -0.14 |
| Bother | 8 | 8 | 76.3 (20.4) | 76.6 (41-100) | 4 | 85.9 (12.2) | 87.5 (68-100) | 5 | 11.3 (23.4) | 6.3 (-10;47) | 0.55 |
| Bowel-summary | 10 | 9 | 78.9 (25.5) | 90.0 (34-100) | 5 | 79.6 (15.0) | 82.5 (64-95) | 5 | 0.8 (36.4) | 0.8 (-36;58) | 0.03 |
| Function | 4 | 9 | 78.9 (23.7) | 87.5 (33-100) | 4 | 87.0 (14.4) | 90.6 (67-100) | 4 | 5.2 (35.1) | 1.0 (-33;52) | 0.22 |
| Bother | 6 | 9 | 78.9 (28.1) | 91.7 (29-100) | 5 | 76.7 (15.8) | 79.2 (58-92) | 5 | -1.7 (42.1) | 0.0 (-42;63) | -0.06 |
| Sexual-summary | 12 | 7 | 46.4 (14.2) | 50.0 (23-63) | 0 | - | - | 0 | - | - | - |
| Function | 7 | 5 | 27.2 (22.1) | 36.9 (0-52) | 0 | - | - | 0 | - | - | - |
| Bother | 5 | 9 | 71.1 (27.2) | 80 (20-100) | 2 | 60.0 (56.6) | 60 (20-100) | 2 | 7.5 (81.3) | 7.5 (-50;65) | 0.28 |
| **FACT-Bl-Cys** | | | | | | | | | | | |
| FACT-PWB | 7 | 9 | 23.0 (4.3) | 23.0 (15-28) | 5 | 24.8 (1.8) | 26.0 (22-26) | 5 | 1.4 (5.0) | 3.0 (-5;8) | 0.33 |
| FACT-SWB | 7 | 9 | 19.2 (5.5) | 21.0 (7-27) | 5 | 15.6 (6.2) | 17.0 (6-22) | 5 | -1.7 (1.8) | -1.0 (-5;0) | -0.31 |
| FACT-EWB | 6 | 9 | 20.1 (1.8) | 19.2 (18-23) | 4 | 21.3 (1.7) | 21.5 (19-23) | 4 | 1.5 (3.8) | 3.0 (-4;4) | 0.83 |
| FACT-FWB | 7 | 9 | 16.2 (6.2) | 17.0 (8-26) | 5 | 14.6 (3.8) | 13.0 (11-20) | 5 | 1.0 (2.1) | 1.0 (-2;3) | 0.16 |
| Bl-Cys domain | 15 | 9 | 37.2 (9.0) | 40.0 (19-48) | 5 | 43.0 (2.7) | 45.0 (40-45) | 5 | 6.0 (9.0) | 5.0 (-5;16) | 0.67 |
| **EQ-5D** | | | | | | | | | | | |
| EQ-5D-5L | 5 | 9 | 0.85 (0.16) | 0.86 (1;1) | 5 | 0.84 (0.11) | 0.88 (1;1) | 5 | -0.01 (0.14) | 0.02 (0;0) | -0.06 |
| EQ-VAS | 1 | 9 | 71.2 (12.4) | 75.0 (51-86) | 8 | 33.8 (36.7) | 27.5 (0-81) | 8 | -35.6 (30.1) | -33.0 (-75;0) | -2.87 |
