# Supplementary material for: Translation and validation of two disease-specific patient-reported outcome measures (Bladder Cancer Index and FACT-Bl-Cys) in Dutch bladder cancer patients
Source: J Patient Rep Outcomes. 2019 Sep 14;3:62. doi: 10.1186/s41687-019-0149-7 (PMC6745039; doi:10.1186/s41687-019-0149-7)
Supplement: Supplementary file 3 — Change in scores between T0 (baseline) and T3 (90 days post-operative) of the measures in relation responsiveness properties. (DOCX 26 kb) [file 41687_2019_149_MOESM3_ESM.docx]

**Additional file 3**

Change in scores between T0 (baseline) and T3 (90 days post-operative) of the measures in relation responsiveness properties

|  | **No change (≤5 points) in EQ-VAS score in T0 and T3**^*^ | | | | | | **Improvement of ≥10 in EQ-VAS score in T0 and T3**^†^ | | | | | | | | **Decline of ≥10 in EQ-VAS score in T0 and T3**^‡^ | | | | | | | |
| --- | --- | --- | --- | --- | --- | --- | --- | --- | --- | --- | --- | --- | --- | --- | --- | --- | --- | --- | --- | --- | --- | --- |
| **Domains** | **Valid population (n)** ^#^ | **T0**  **Mean (SD)** | **T3**  **Mean (SD)** | **Mean change**  **T0-T3 (SD)** | **Effect Size (ES)**^§^ | **Correlation changes (r)**^¶^ | **Valid population (n)** ^#^ | **T0**  **Mean (SD)** | **T3**  **Mean (SD)** | **Mean change**  **T0-T3 (SD)** | **Effect Size (ES)**^§^ | **Correlation changes (r)**^¶^ | **Hypotheses confirmed?** | | **Valid population (n)** ^#^ | **T0**  **Mean  (SD)** | **T3**  **Mean  (SD)** | **Mean change**  **T0-T3 (SD)** | **Effect Size (ES)**^§^ | **Correlation changes (r)**^¶^ | **Hypotheses confirmed?** | |
|  |  |  |  |  |  |  |  |  |  |  |  |  | **ES**^a^ | **r**^b^ |  |  |  |  |  |  | **ES**^a^ | **r**^b^ |
| **EQ-VAS** | 68 | 83 (12) | 83 (13) | 0 (3) | 0.00 | 1.00 | 55 | 54 (19) | 79 (11) | 25 (16) | 1.32 | 1.00 | NA. | NA. | 89 | 75 (17) | 25 (32) | -50 (30) | -2.94 | 1.00 | NA. | NA. |
| **BCI** |  |  |  |  |  |  |  |  |  |  |  |  |  |  |  |  |  |  |  |  |  |  |
| Urinary domain | 55 | 85 (16) | 86 (17) | 1 (22) | 0.06 | 0.10 | 42 | 75 (22) | 82 (19) | 8 (22) | 0.36 | 0.17 | **Yes** | No | 32 | 85 (16) | 79 (17) | -7 (20) | -0.44 | -0.12 | **Yes** | No |
| Function | 51 | 88 (20) | 79 (31) | -9 (39) | -0.45 | 0.00 | 43 | 83 (27) | 72 (37) | -12 (43) | -0.44 | -0.13 | No | No | 29 | 83 (27) | 68 (35) | -15 (40) | -0.56 | -0.19 | **Yes** | No |
| Bother | 52 | 84 (17) | 89 (13) | 6 (18) | 0.35 | 0.04 | 38 | 71 (43) | 85 (14) | 15 (25) | 0.35 | 0.29 | No | No | 31 | 84 (19) | 85 (13) | -1 (17) | -0.05 | -0.05 | No | No |
| Bowel domain | 57 | 91 (10) | 83 (16) | -9 (18) | -0.90 | 0.32 | 47 | 81 (18) | 80 (15) | -1 (17) | -0.06 | 0.42 | No | **Yes** | 40 | 86 (16) | 80 (21) | -7 (23) | -0.44 | 0.11 | No | No |
| Function | 53 | 89 (14) | 83 (19) | -6 (22) | -0.43 | 0.24 | 46 | 82 (19) | 84 (14) | 1 (17) | 0.05 | 0.25 | No | No | 38 | 88 (14) | 83 (19) | -5 (23) | -0.36 | -0.18 | No | No |
| Bother | 57 | 92 (12) | 83 (17) | -9 (19) | -0.75 | 0.29 | 47 | 80 (19) | 78 (19) | -2 (21) | -0.11 | 0.39 | No | **Yes** | 39 | 86 (19) | 78 (21) | -8 (26) | -0.42 | 0.18 | No | No |
| Sexual domain | 15 | 49 (20) | 39 (18) | -17 (16) | -0.85 | -0.01 | 20 | 45 (20) | 35 (18) | -10 (19) | -0.50 | 0.23 | No | No | 10 | 52 (20) | 39 (17) | -22 (22) | -1.10 | -0.14 | **Yes** | No |
| Function | 14 | 32 (19) | 22 (18) | -13 (19) | -0.68 | 0.01 | 17 | 30 (21) | 23 (15) | -10 (17) | -0.48 | 0.25 | No | No | 12 | 36 (20) | 22 (17) | -25 (20) | -1.25 | -0.36 | No | **Yes** |
| Bother | 43 | 66 (31) | 53 (33) | -11 (41) | -0.35 | 0.06 | 34 | 64 (31) | 53 (30) | -9 (32) | -0.29 | 0.04 | No | No | 28 | 72 (29) | 47 (30) | -21 (39) | -0.72 | 0.28 | No | No |
| **FACT-Bl-Cys** |  |  |  |  |  |  |  |  |  |  |  |  |  |  |  |  |  |  |  |  |  |  |
| FACT-PWB | 66 | 26 (2) | 25 (2) | 0 (2) | 0.00 | 0.54 | 52 | 21 (5) | 25 (2) | 4 (5) | 0.80 | 0.51 | **Yes** | **Yes** | 42 | 23 (5) | 22 (5) | -2 (5) | -0.40 | 0.44 | **Yes** | **Yes** |
| FACT-SWB | 65 | 22 (4) | 21 (5) | -2 (4) | -0.50 | 0.26 | 52 | 21 (6) | 21 (5) | -1 (6) | -0.17 | 0.20 | No | No | 41 | 22 (4) | 22 (3) | 0 (4) | 0.00 | 0.24 | No | No |
| FACT-EWB | 61 | 18 (5) | 21 (4) | 3 (4) | 0.60 | 0.30 | 49 | 17 (5) | 21 (3) | 4 (5) | 0.80 | 0.40 | **Yes** | **Yes** | 40 | 18 (4) | 20 (4) | 1 (3) | 0.25 | 0.38 | No | **Yes** |
| FACT-FWB | 64 | 19 (5) | 20 (4) | 1 (4) | 0.20 | 0.42 | 51 | 14 (6) | 19 (5) | 4 (5) | 0.67 | 0.47 | **Yes** | **Yes** | 40 | 18 (5) | 17 (5) | -2 (5) | -0.40 | 0.31 | **Yes** | **Yes** |
| Bl-Cys domain | 64 | 43 (9) | 45 (7) | 2 (10) | 0.22 | 0.13 | 50 | 37 (11) | 43 (9) | 6 (12) | 0.55 | 0.44 | **Yes** | **Yes** | 41 | 42 (9) | 42 (8) | -2 (9) | -0.22 | 0.42 | **Yes** | **Yes** |
| FACT-Bl-Cys total | 59 | 129 (18) | 132 (15) | 3 (15) | 0.17 | 0.39 | 45 | 109 (24) | 130(17) | 18 (20) | 0.75 | 0.62 | **Yes** | **Yes** | 40 | 123 (22) | 122 (21) | -4 (19) | -0.18 | 0.53 | **Yes** | **Yes** |

^*^No change between T0 and T3 for patients in EQ-VAS score (plus or minus ≤5 points); ^†^Change between T0 and T3 for patients with an improved EQ-VAS score of ≥10 points; ^‡^Change between T0 and T3 for patients with an deteriorated EQ-VAS score of ≥10 points; ^#^The number of patients that completed enough items to calculate a total score; ^§^Formula assessed for Effect Size (Cohens d): ChangeT3T0_Mean_/T0_SD_; ^¶^Spearman correlation (r) between change score EQ-VAS and change score BCI and FACT-Bl-Cys (T0 and T3); ES = Effect size (in Cohens d); r = Spearman correlation;
^a^ Hypothesis = in patients with an improved or declined EQ-VAS score (≥10 points), we hypothesized to find a larger effect size, in comparison to patients that did not show a change in EQ-VAS (≤5 points).

^b^ Hypothesis = in patients with an improved or declined EQ-VAS score (≥10 points), we hypothesized to find moderate correlations of ≥0.30 between change in EQ-VAS and the change in BCI and FACT-Bl-Cys values;
